# Supplementary material for: Development of a Positive Psychology Well-Being Intervention in a Community Pharmacy Setting
Source: Pharmacy (Basel). 2023 Jan 11;11(1):14. doi: 10.3390/pharmacy11010014 (PMC9844442; doi:10.3390/pharmacy11010014)
Supplement: Supplementary file 1 [file pharmacy-11-00014-s001.zip › pharmacy-2072371-supplementary.pdf]

Supplementary Material 1

Literature Search Strategy

The aim of the literature review was to investigate the effectiveness of PPIs in community-based samples. Three specific research objectives were:

1. To investigate the short-term effects (post-intervention, 1-week, 2-weeks, and 1-month) of PPIs delivered in community-based samples for enhancing well-being,
2. To investigate the sustainability of effect of PPIs (3-, 6-months) delivered in community-based samples for enhancing well-being,
3. To investigate the potential factors, such as recruitment strategies, retention, participants, and characteristics of interventions that support the effectiveness of PPIs.

Methods

At the first phase, literature selection was based on title, abstract and keywords and then selection was based on the full text. The PICO (Participant Intervention, Comparison, Outcomes) [38] framework was applied to investigate studies that met the inclusion criteria.

Inclusion criteria

The inclusion criteria were as follows:

- Participant: Participants recruited in Western Society populations and from community-based settings only.
- Intervention: Inclusion of at least one of the following Positive Psychology Interventions (or a theoretical model) (search order):
    - *PERMA* (Positive Emotions, Engagement, Relationships, Meaning, Accomplishment)
    - *Best Possible Selves*
    - *Three Good Things*
    - *Using Your Signature Strengths in a New Way*
    - *Identifying Your Signature Strengths*
- Comparison: At least one comparison group i.e., *Early Memories* or alternative control group activity
- Outcomes: Well-being is measured from pre-intervention to post-intervention. Any measure which includes items to assess mood, psychological well-being and/or subjective well-being [71,91].

Study Design

No strict inclusion criteria were applied for the type of study design.

Exclusion criteria

Studies were excluded if:

- Participants were recruited from the following settings:
    - Education, Workplace, Military and Healthcare.
- The following activities formed part of the intervention which fall outside the definition of PPIs:
    - Physical activity, Mindfulness, Meditation, Forgiveness and Life therapy
- The following reviews of the literature were excluded if they were:
    - Literature reviews, Critical reviews, Scoping review, Systematic reviews and Meta-analyses

Search Engines and Keywords

Searches were carried out in PsychInfo, PubMed and Scopus covering the period from 2011 to March 2018 [Step 1], then repeated to October 2019 [Step 2]. The PERMA well-being model [20] was published in 2011, therefore, the review was conducted from 2011 for the most recent PPIs.

The following keywords were used to explore *PERMA-based* interventions: "PERMA" in combination with "positive psychology intervention" or "PPI".
For the *Best Possible Selves* Intervention, text words used: "BPS" or "Best possible selves" or "best possible self" in combination with "PPI" and "positive psychology intervention".
For *Three Good Things* intervention text words were limited to: "three good things" or "TGT" in combination with "PPI" and "Positive Psychology Intervention".
For *Character Strengths*-based interventions, text words were limited to: "character strengths" or "character strengths and virtue" or "character strengths and virtues" or "character strengths in a new way" or "utilising your signature strengths in a new way" in combination with "PPI" and "Positive Psychology Intervention".
"Community" and "Public" were the text words used to explore the target population.

Table S1: Search Strategy and Keywords used for Critical Literature Review

**Search 1 – Keywords**	**Search 2 – Keywords**	**Search 3 – Keywords**	**Search 4 – Keywords**
"PERMA"<br>and<br>"positive psychology intervention"<br>and<br>"community"	"three good things"<br>or<br>"TGT"<br>and<br>"positive psychology intervention"	"best possible selves"<br>or<br>"BPS"<br>and<br>"positive psychology intervention" and<br>"community"	"character strengths in a new way"<br>or<br>"character strengths and virtues"<br>and<br>"positive psychology intervention"

The search was restricted to research articles and peer-reviewed studies in the English language.
Titles and abstracts were assessed for relevance and duplicates were removed.
Community was only added to specific searches due to the null result of studies when this keyword was added to the initial search.
Studies which did not include "community" were manually searched focusing on how participants were recruited.

Study Description

Studies were scanned following the PICOS [38] framework to record participant characteristics (number of participants in the study, average age, gender), intervention (type, sessions, duration, type of delivery), comparison group, outcomes (well-being measurement and timeframe) and study design (research design, quality of study, attrition rate at post-intervention).

Statistical Analysis

The p value was set at the class 0.05 threshold, with $p<0.05$ indicating a statistical significance and $p>0.05$ resulting in no statistical significance difference [40,41]. Studies were investigated to understand the effectiveness of PPIs by focusing on whether the intervention had a statistically significant effect on well-being at post-intervention, 1-week, 2-weeks, and 1-month follow up. Additionally, studies were investigated to understand whether PPIs had a statistically significant sustainable effect on well-being 3-, and/or 6-months. Finally, the standardised mean effect sizes were calculated utilising the Wilson Practical Meta-Analysis Effect Size Calculator to understand the magnitude of intervention effectiveness across different timepoints (i.e., post-intervention, 1-week, 2-weeks, 1-, 3- and/or 6-months) on well-being. Effect sizes were assessed as small (0.2), medium (0.5) and large (0.8) [40,41]. Recruitment strategies, retention, participant, and intervention characteristics were explored to understand their potential influence on the effectiveness of PPIs.

Supplementary Material 2

PRISMA Flow Chart

A total of nine studies were identified. The PRISMA selection process is illustrated in Figure .1.

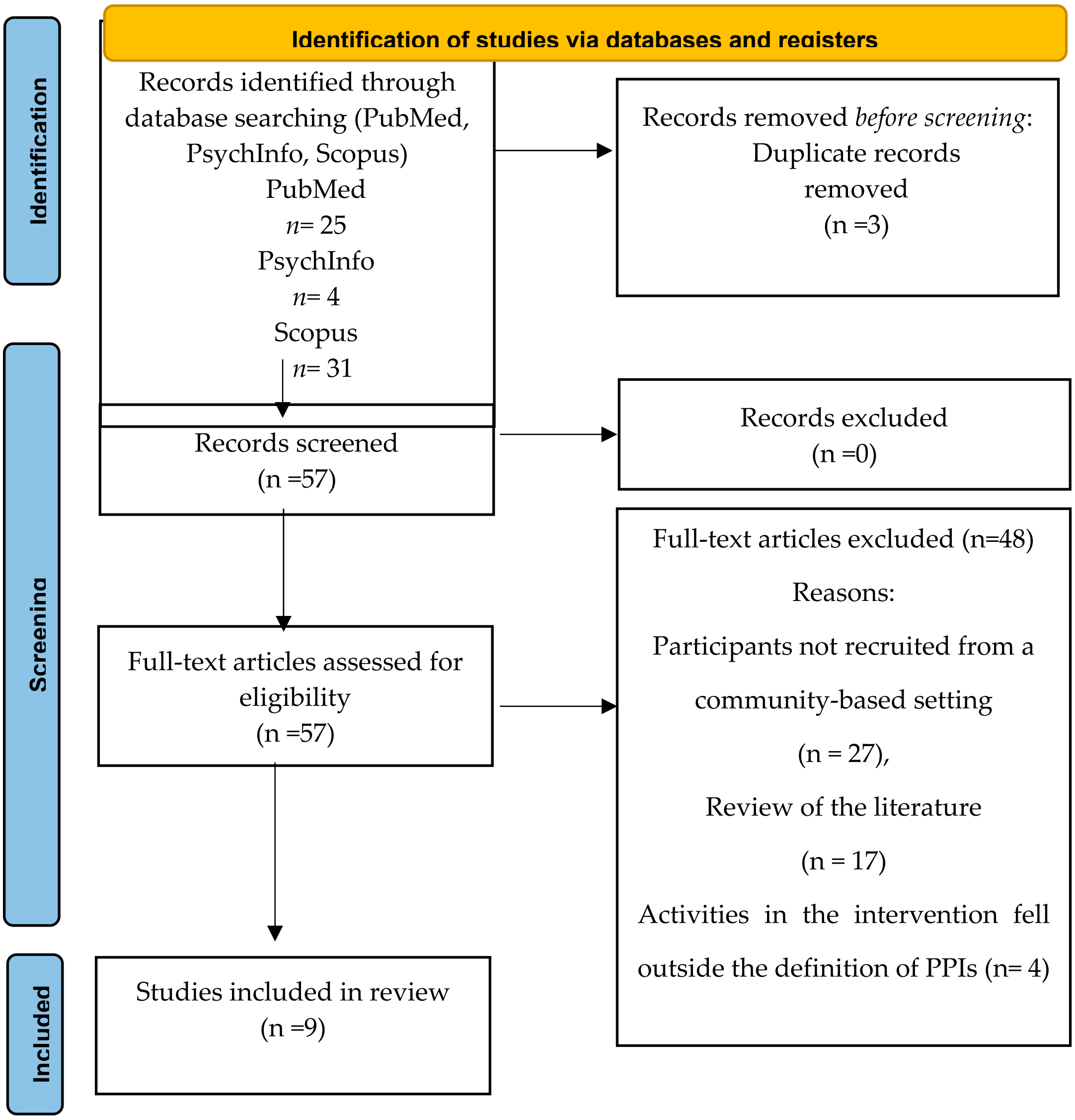

Figure S1. Flow diagram of studies that were identified using the search term and strategy, articles screened for eligibility included/ excluded with reasons following the PRISMA guidelines.

Supplementary Material 3

Table S1. Description of Positive Psychology Interventions in Community-Based Samples (n=9 studies)

Author, Year, Country(ies)	Research Design	Interven-tion(s) Type	Interven-tion Ses-sions, Du-ration, Type of Delivery	Compari-son (Control) Group Type	Well-be-ing Measure	(N=)	Attrition Rate % (Post-Intervention)	Average Age	Female (%)	Quality of Methodology
[42] Gander, Proyer and Ruch (2016), Germany / Switzerland	RCT	*PERMA*	7, 1-week, Self-ad-ministered online	Early Memo-ries	AHI	1624	55%	Middle Age	79.2%	6
[48] Proyer et al (2015), Switzerland	RCT	*Signature Strengths, Lesser Strengths*	7, 1-week, Self-administer ed online	Early Memorie s	AHI	720	52%	Middle Age	83.7%	5
[43] Gander, Proyer and Ruch (2017), Switzerland / Germany	RCT	*PERMA*	7,1-week, Self-administer ed online	Placebo Control Exercise	SWLS	112	60%	Young Age	62.7%	5
[47] Mongrain and Anselmo-Mattews (2012), United Kingdom	RCT	*Three Good Things, Signature Strengths*	7, 1-week, Self-administer ed online	Early Memorie s	SHI	1447	24%	Young Age	83%	4
[49] Proyer et al (2014), Switzerland	RCT	*Signature Strengths, Gratitude Visit, Three Good Things,*	7, 1-week, Self-administer ed online	Early Memorie s	AHI	510	32%	Middle Age	100%	4
[45] Odou-Vella and Brodrick (2013), Australia	RCT	*Three Good Things, Best Possible Selves*	7,1-week, Self-administer ed online	No Activity Control Group	PANAS WEBWBS	210	18%	Young Age	78%	3
[46] Seear and Vella-Brodrick (2013), Australia	RCT	*Three Good Things, Best Possible Selves*	7, 1-week, Self-administer ed online	No Activity Control Group	WEBWBS PANAS	211	16%	Young Age	75.3%	3
[44] Greenawalt et al (2018), USA	Cross-Sectional Longitudi nal Study	*PERMA*	8, 8-weeks, Face to Face, Group-administer ed	No Activity Control Group	SWLS SHS	52	0%	Older Age	88.5%	2
[50] Woodworth et al (2016), Australia	N-1 Counterba lanced Design	*Three Good Things, Signature Strengths, Gratitude Visit*	1, 9-10weeks, Self-administer ed online	No Activity Control Group	PANAS	15	0%	Middle Age	73.3%	2

Key.
Positive and Negative Affect Schedule= PANAS; Warwick-Edinburgh Mental Well-Being= WEBWBS; Authentic Happiness Index= AHI; Epidemiologic Studies Depression Scale= CES-D; Satisfaction with Life Scale= SWLS; Orientations to Happiness Questionnaire= OTH; Relationship Scale= REL; Accomplishment Scale= ACC; Subjective Happiness Scale= SHS; Depression Scale= PHQ-9; SHI= Steen Happiness Index

Methodological Quality

Three studies were rated as high quality [45, 46, 62], four as medium quality [53, 54, 61, 63] and two as low quality [47, 60]. Table 2 shows the breakdown of scores for each criterion for the assessment of quality of the methodology of the nine studies.

Table S2. Assessment of Methodological Quality of Studies (n=9 studies)

Authors (Year) / Criterion	1	2	3	4	5	6	Total
[42] Gander, Proyer and Ruch (2016)	1	1	1	1	1	1	6
[48] Proyer et al (2015)	1	1	1	1	1	0	5
[43] Gander, Proyer and Ruch (2017)	1	1	1	1	1	0	5
[47] Mongrain and Anselmo-Mattews (2012)	1	-	1	1	1	0	4
[49] Proyer et al (2014)	1	-	1	1	0	0	4
[45] Odou and Vella-Brodrick (2013)	1	-	1	1	0	0	3
[46] Seear and Vella-Brodrick (2013)	1	0	1	1	0	0	3
[44] Greenwalt et al (2018)	0	0	1	1	0	0	2
[50] Woodworth et al (2016)	0	0	1	1	0	0	2

Key:

Index; 1= yes; 0= no; - = missing data

Criterion: 1= Adequacy of randomization concealment, 2= Blinding of subjects to the condition (blinding of assessors was not applicable in most cases), 3= Baseline comparability, 4= Power analysis: is there an adequate power analysis and/or are there at least 50 participants in the analysis? 5=Completeness of follow up data: clear attrition analysis and loss to follow up < 50%, 6= Handling of missing data: the use of intention-to-treat analysis.

Rating: High= 5-6; Medium= 3-4; Low= 0-2

Table S3. Effectiveness of Positive Psychology Interventions in Community-Based Samples on Well-being over Time (n=9 studies)

Author (Year)	Quality of Methodologies	Intervention Sessions, Duration, Type	Well-being Measurement	Effectiveness on Well-being over Time				
				**Post**	**2W**	**1M**	**3M**	**6M**
[42] Gander, Proyer and Ruch (2016)	High	7,1-week, PERMA	AHI	*	-	*	*	*
[48] Proyer et al (2015)	High	7,1-week, *Signature Strengths*	AHI	*	*	*	*	NS
[43] Gander, Proyer and Ruch (2017)	Medium	7,1-week, *PERMA*	SWLS	*	NS	NS	-	-
[43] Gander, Proyer and Ruch (2017)	Medium	7,1-week, *PERMA*	PANAS (PA), PANAS (NA)	*	NS	NS	-	-
[47] Mongrain and Anselmo-Mattews (2012)	Medium	7,1-week, *Three Good Things*	SHI	*	*	*	*	*
[47] Mongrain and Anselmo-Mattews (2012)	Medium	7,1-week, *Signature Strengths*	SHI	*	*	*	*	*
[49] Proyer et al (2014)	Medium	7,1-week, *Signature Strengths*	AHI	*	-	*	*	*
[49] Proyer et al (2014)	Medium	7,1-week, *Three Good Things*	AHI	*	-	NS	NS	NS
[49] Proyer et al (2014)	Medium	7,1-week, *Gratitude Visit*	AHI	*	-	*	NS	NS
[45] Odou and Vella-Brodrick (2013)	Medium	7,1-week, *Best Possible Selves*	PANAS (PA), PANAS (NA)	NS, *	NS, NS	-	-	-
[45] Odou and Vella-Brodrick (2013)	Medium	7,1-week, *Best Possible Selves*	WEMWBS	NS	NS	-	-	-
[45] Odou and Vella-Brodrick (2013)	Medium	7,1-week, *Three Good Things*	WEMWBS	NS	NS	-	-	-
[45] Odou and Vella-Brodrick (2013)	Medium	7,1-week, *Three Good Things*	PANAS (PA), PANAS (NA),	NS	NS	-	-	-
[46] Seear and Vella-Brodrick (2013)	Medium	7,1-week, *Three Good Things*	WEMWBS	NS	NS	-		
[46] Seear and Vella-Brodrick (2013)	Medium	7,1-week, *Three Good Things*	PANAS (PA), PANAS (NA),	NS, *	NS, NS	-	-	-

[46] Seear and Vella-Brodrick (2013)	Medium	7,1-week, *Best Possible Selves*	WEMWBS	NS,	NS,	-	-	-
[46] Seear and Vella-Brodrick (2013)	Medium	7,1-week, *Best Possible Selves*	PANAS (PA),<br>PANAS (NA),	NS,<br>*	NS,<br>NS	-	-	-
[44] Greenwalt et al (2018)	Low	8, 8-weeks, *PERMA*	SWLS, SHS	*	-	-	-	-
[50] Woodworth et al (2016)	Low	1, 9-10weeks, *Three Good Things, Gratitude Visit*	PANAS (PA),<br>PANAS (NA),	NS,<br>NS	-	-	-	-
[50] Woodworth et al (2016)	Low	1, 9-10weeks, *Signature Strengths*	PANAS (PA),<br>PANAS (NA),	*,<br>NS	-	-	-	-

Key:
Negative Affect Schedule= PANAS; Positive and Negative Affect Schedule, Positive Affect= PANAS (PA), Positive and Negative Affect Schedule, Negative Affect= PANAS (NA); Warwick-Edinburgh Mental Well-Being= WEBWBS; Authentic Happiness Index= AHI; Epidemiologic Studies Depression Scale= CES-D; Satisfaction with Life Scale= SWLS; Orientations to Happiness Questionnaire= OTH; Relationship Scale= REL; Accomplishment Scale= ACC; Subjective Happiness Scale= SHS; Depression Scale= PHQ-9; SHI= Steen Happiness Index ; * = Statistically Significant, NS= Not Statistically Significant p<0.05, - = Well-being measurement not taken at timepoint.

pharmacy MDPI

Table S4. Mean Effect Size (Cohen's d) for Positive Psychology Interventions compared to Control Groups over Time (n=9 studies)

**Author (Year)**	**Intervention Sessions, Duration, Type**	**Well-being Measurement**	**Mean Effect Size over time on well-being**				
			**Post**	**2W**	**1M**	**3M**	**6M**
[42] Gander, Proyer and Ruch (2016)	7, 1-week, PERMA	AHI	0.2	-	0.1	0.2	0.1
[48] Proyer et al (2015)	7, 1-week, *Signature Strengths*	AHI	0.0	-	0.1	0.0	0.1
[43] Gander, Proyer and Ruch (2017)	7, 1-week, *PERMA*	SWLS	0.032	0.2	0.4	-	-
[43] Gander, Proyer and Ruch (2017)	7, 1-week, *PERMA*	PANAS (PA),<br>PANAS (NA)	0.1<br>-0.2	0.1<br>-0.1	0.1<br>-0.3	-	-
[47] Mongrain and Anselmo-Mattews (2012)	7, 1-week, *Three Good Things*	SHI	0.0	-	0.1	0.0	0.2
[47] Mongrain and Anselmo-Mattews (2012)	7, 1-week, *Signature Strengths*	SHI	0.1	-	0.1	0.1	0.3
[49] Proyer et al (2014)	7, 1-week, *Signature Strengths*	AHI	0.4	-	0.7	0.0	0.1
[49] Proyer et al (2014)	7, 1-week, *Three Good Things*	AHI	0.4	-	0.3	0.1	0.1
[49] Proyer et al (2014)	7, 1-week, *Gratitude Visit*	AHI	0.1	-	0.1	0.1	0.1
[45] Odou and Vella-Brodrick (2013)	7, 1-week, *Best Possible Selves*	PANAS (PA)<br>PANAS (NA)	2.2<br>-3.01	3.0,<br>-1.0	-	-	-
[45] Odou and Vella-Brodrick (2013)	7, 1-week, *Best Possible Selves*	WEMWBS	0.6	0.8	-	-	-
[45] Odou and Vella-Brodrick (2013)	7,1-week, *Three Good Things*	WEMWBS	0.4	0.6	-	-	-
[45] Odou and Vella-Brodrick (2013)	7,1-week, *Three Good Things*	PANAS (PA)<br>PANAS (NA),	2.2<br>-3.2	2.3,<br>-2.5	-	-	-
[46] Seear and Vella-Brodrick (2013)	7,1-week, *Three Good Things*	WEMWBS	0.6	0.4	-		
[46] Seear and Vella-Brodrick (2013)	7,1-week, *Three Good Things*	PANAS (PA)<br>PANAS (NA)	0.4,<br>-0.7	0.6,<br>-1.0	-	-	-
[46] Seear and Vella-Brodrick (2013)	7,1-week, *Best Possible Selves*	WEMWBS	0.5	0.7	-	-	-
[46] Seear and Vella-Brodrick (2013)	7,1-week, Best Possible Selves	PANAS (PA)<br>PANAS (NA)	0.3<br>-0.6	0.6,<br>-0.4	-	-	-
[44] Greenwalt et al (2018)	8, 8-weeks, PERMA	SWLS,<br>SHS	0.1,<br>0.9	-	-	-	-

Key: Positive and Negative Affect Schedule= PANAS; Positive and Negative Affect Schedule, Positive Affect= PANAS (PA), Positive and Negative Affect Schedule, Negative Affect=PANAS (NA);Warwick-Edinburgh Mental Well-Being=

WEBWBS; Authentic Happiness Index= AHI; Epidemiologic Studies Depression Scale= CES-D; Satisfaction with Life Scale= SWLS; Orientations to Happiness Questionnaire= OTH; Relationship Scale= REL; Accomplishment Scale= ACC; Subjective Happiness Scale= SHS; Depression Scale= PHQ-9; SHI= Steen Happiness Index; - = Well-being measurement not taken at timepoint.

Supplementary Material 4

Version 3 of The *Prescribing Happiness*, Session 1

Prescribing Happiness Intervention

A PHD STUDY-

CARDIFF METROPOLITAN UNIVERSITY,

SCHOOL OF SPORT AND HEALTH SCIENCES,

IN COLLABORATION WITH

THE HEALTH DISPENSARY PHARMACY AND WELLNESS CLINIC, NEATH

Ms Jennifer Ward (PhD Researcher & **Wellbeing Facilitator)

Dr Delyth H James (Director of studies)

Ms Alison Sparkes (Company Partner)

Dr Paul Hewlett (Supervisor)

Dr Amie-Louise Prior (Supervisor)

© Jennifer Ward, Alison Sparkes, Paul Hewlett, Amie-Louise Prior, Delyth James

Prescribing Happiness Session One

'Character Strengths'

Welcome to the first session of the 'Prescribing Happiness' Intervention at The Health Dispensary Pharmacy and Wellness Clinic. This session aims to facilitate and support you on identifying your **Character Strengths**.

Our Character Strengths are the qualities that come most naturally to us, they are the things which we are good at. Identifying and using our Character Strengths help us to manage stressors and challenges we may face in life; they help us to improve our relationships.

They promote positive well-being, allow us to become more engaged and mindful in our lives and they give us a sense of purpose, meaning and encourage us to feel more valued and fulfilled in life.

© Jennifer Ward, Alison Sparkes, Paul Hewlett, Amie-Louise Prior, Delyth James

Activity One: Introduce Yourself

We'd like you to think about your strengths, your qualities, things which you are good at in all areas of your life; home, relationships, hobbies or at work. If you are struggling to think about your strengths, maybe think about what your friends, family or others have complimented you on.

Write down **5 things** about yourself…

**1.**
**2.**
**3.**
**4.**
**5.**

Ward et al, 2019.

Tip: Don't write your age, name, job, focus on everything you are good. Don't be afraid to compliment yourself.

© Jennifer Ward, Alison Sparkes, Paul Hewlett, Amie-Louise Prior, Delyth James

What are the 24 Character Strengths?

We all have our own strengths; these strengths play an important role in our life. Our strengths are unique, everyone has different strengths and weaknesses and it is important that we recognise what we are good at and how to best use them.

Appreciation of Beauty and Excellence	Forgiveness	Kindness	Perseverance	Bravery	Gratitude
Leadership	Prudence	Connection/ Purpose	Hope/Optimism	Love	Self-Control
creativity	Humility/ Modesty	Love of Learning	Social Intelligence	Curiosity	Humour
Open-Mindedness	Teamwork/ Citizenship	Fairness	Integrity	Perspective	Zest/ Enthusiasm

Seligman and Peterson (2004).

Tip: If you have any questions or are unsure of any of the meaning of the character strengths, please feel free to ask.

© Jennifer Ward, Alison Sparkes, Paul Hewlett, Amie-Louise Prior, Delyth James

What are your 5 Signature Strengths?

I'd like you to think of things about yourself, using the 24-character strengths please take a moment and write down your top five signature strengths.

1.

2.

3.

4.

5.

Tip: If you are struggling to think about strengths, maybe think about your friends, family or others have complimented you on

© Jennifer Ward, Alison Sparkes, Paul Hewlett, Amie-Louise Prior, Delyth James

The Prescribing Happiness Diary

The Guidelines:

- In your spare time, please take 10 to 15-minutes to complete your diary
- You are asked to complete **3 diary entries** a week
- The best time is in the evening or after your working day
- Sometimes we experience multiple 'PERMA' elements a day, sometimes we may experience none.
- Please only use **1 Example** from your day and explain what the element was. For each diary entry, please free to go into as much detail as you wish.

© Jennifer Ward, Alison Sparkes, Paul Hewlett, Amie-Louise Prior, Delyth James

What will we do Next Week?

Week One	Week Two	Week Three	Week Four	Week Five	Week Six
**Character Strengths**	**Positive Emotions:** Gratitude and Hope	**Engagement:** Living Mindfully in the Present Moment	**Relationships:** Importance of Social Relationships	**Meaning:** Find your Passion and Values	**Accomplishment:** Planning for the Future

© Jennifer Ward, Alison Sparkes, Paul Hewlett, Amie-Louise Prior, Delyth James

An Example from Week 1 of The *Prescribing Happiness* PERMA-based Diary

Welcome to Your Prescribing Happiness Diary

Contact Us

If you have any queries, questions or want to ask something please get in contact:

Name: Miss Jennifer Ward (PhD Researcher and Wellbeing Facilitator)
Email: jeward@cardiffmet.ac.uk
Location: The Health Dispensary Pharmacy and Wellness Clinic, Neath

© Jennifer Ward, Alison Sparkes, Professor Delyth James

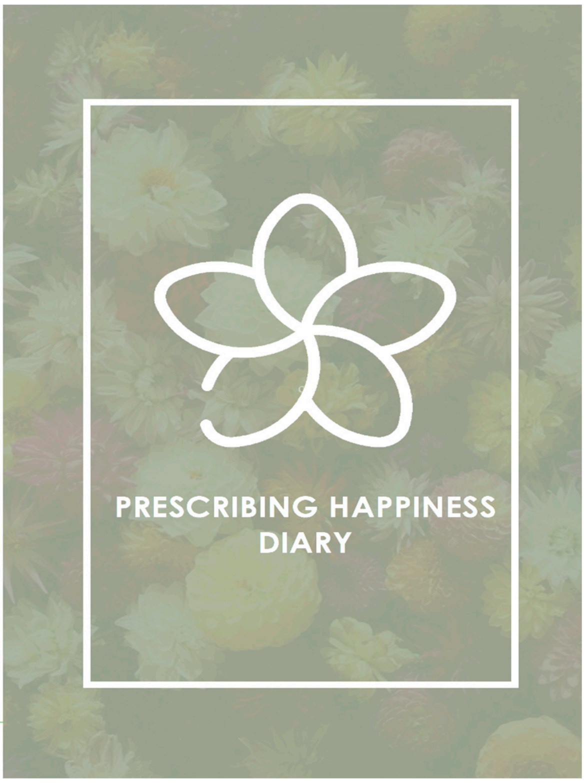

The Prescribing Happiness Diary Guidelines

The Prescribing Happiness Diary is your own personalised and private wellbeing diary that we have designed to support you. It will be completed alongside the workshop sessions with support from the Wellbeing Facilitator.

The Guidelines:

- In your spare time, please take **10 to 15-minutes** to complete your diary
- You are asked to complete **3 Diary Entries** a week
- The best time is in the **Evening or after your Working Day**
- Sometimes we experience multiple 'PERMA' elements a day, sometimes we may experience none.
- Please only use **1 Example** from your day and explain what the element was.
- For each diary entry, please free to go into as much detail as you wish

PERMA Reminder:

Positive Emotions	Engagement	Relationship	Meaning	Accomplishment
'reported feelings of contentment, hope, gratitude, pleasure and joy'<br><br>'reported positive feelings'	'being absorbed and interested in something where you lose track of time'<br><br>'focusing on the here and now rather than the future'	'feeling connected, integrated as part of the community and being cared for and loved for'	'having a direction connecting them to something rather than oneself'<br><br>'feeling what one does is valuable'	'the perseverance towards a goal or future outcome'

© Jennifer Ward, Alison Sparkes, Professor Delyth James

WELCOME TO WEEK ONE

Day	Day: ______________ Date: __________	
PERMA & Your Example	P	
	E	
	R	
	M	
	A	

© Jennifer Ward, Alison Sparkes, Professor Delyth James

WELCOME TO WEEK ONE

Day	Day: ______________ Date: __________	
PERMA & Your Example	P	
	E	
	R	
	M	
	A	

© Jennifer Ward, Alison Sparkes, Professor Delyth James

WELCOME TO WEEK ONE

Day	Day: _______________ Date: ___________	
PERMA & Your Example	P	
	E	
	R	
	M	
	A	

© Jennifer Ward, Alison Sparkes, Professor Delyth James

Supplementary Material 5

Table S1 - TIDier Checklist

**TIDieR Checklist Criteria**	**Item Description**	**Result**
**Name of Intervention**	*Provide the name or a phrase that describes the intervention*	*Prescribing Happiness (P-Hap)*
**Why**	*Describe any rationale, theory, or goal of the elements essential to the intervention*	To design an intervention that can be delivered in a community pharmacy setting. The aim of the PPI is to elicit positive emotions, engagement, a sense of relationships, meaning and accomplishment (PERMA) to promote well-being in community members.
**What**	***Materials:*** *Describe any physical or informational materials used in the intervention, including those provided to participants or used in intervention delivery or in training of intervention providers. Provide information on where the materials can be accessed (such as online appendix, URL)*	A total of six worksheets were developed. One worksheet to be completed in each workshop. The full version of the intervention and the PERMA-based diary is described.
	Procedures: *Describe each of the procedures, activities, and/or processes used in the intervention, including any enabling or support activities*	The rationale for included intervention activities, procedures and processes is presented in the paper. Table 3 displays and describes the main activities included in the intervention.
**Who provided**	*For each category of intervention provider (such as psychologist, nursing assistant), describe their expertise, background, and any specific training given*	The intervention was delivered by the Well-being Facilitator (JLW) who was also the researcher. The researcher undertook this role as part of a PhD and has a BSc in Sport & Exercise Science, MSc in Psychology. JLW undertook additional courses in positive psychology and motivational interviewing (MI). JLW also holds a Mental Health First Aid training certificate.
**How:**	*Describe the modes of delivery (such as face to face or by some other mechanism, such as internet or telephone) of the intervention and whether it was provided individually or in a group*	One-to-one session delivered face-to-face

**Where**	*Describe the type(s) of location(s) where the intervention occurred, including any necessary infrastructure or relevant features*	The Health Dispensary Pharmacy (a community pharmacy) and Wellness Clinic, Consultation Room 3 based in Neath, South Wales
**When and how much**	*Describe the number of times the intervention was delivered and over what period of time including the number of sessions, their schedule, and their duration, intensity, or dose*	Workshop sessions were delivered once a week for a 45-minute period for 6-weeks. Session scheduling was based on the participant's availability and were offered Monday to Saturday ween 9:00am to 5:30pm. A PERMA-based diary was to be completed 3 days a week for 10 to 15 minutes outside the workshop sessions for a period of 6-weeks.
***Tailoring:**	*If the intervention was planned to be personalised, titrated or adapted, then describe what, why, when, and how*	Not applicable to this intervention
***Modifications:**	*If the intervention was modified during the course of the study, describe the changes (what, why, when, and how)*	Described in Table 2
**How well:**	*Planned: If intervention adherence or fidelity was assessed, describe how and by whom, and if any strategies were used to maintain or improve fidelity, describe them*	Not applicable in this phase of development
	**Actual: If intervention adherence or fidelity was assessed, describe the extent to which the intervention was delivered as planned*	Not applicable in the phase of development
